# Relationship Between Social Motivation in Children With Autism Spectrum Disorder and Their Parents

**DOI:** 10.3389/fnins.2021.660330

**Published:** 2021-05-26

**Authors:** Mirko Uljarević, Thomas W. Frazier, Booil Jo, Jennifer M. Phillips, Wesley Billingham, Matthew N. Cooper, Antonio Y. Hardan

**Affiliations:** ^1^Melbourne School of Psychological Sciences, University of Melbourne, Melbourne, VIC, Australia; ^2^School of Psychology and Public Health, La Trobe University, Melbourne, VIC, Australia; ^3^Department of Psychology, John Carroll University, University Heights, OH, United States; ^4^Department of Psychiatry and Behavioral Sciences, Stanford University, Stanford, CA, United States; ^5^Telethon Kids Institute, University of Western Australia, Perth, WA, Australia

**Keywords:** social motivation, familiality, broader autism phenotype, autism spectral disorder, heterogeneity

## Abstract

Impairment in social motivation (SM) has been suggested as a key mechanism underlying social communication deficits observed in autism spectrum disorder (ASD). However, the factors accounting for variability in SM remain poorly described and understood. The current study aimed to characterize the relationship between parental and proband SM. Data from 2,759 children with ASD (*M*_*age*_ = 9.03 years, SD_*age*_ = 3.57, 375 females) and their parents from the Simons Simplex Collection (SSC) project was included in this study. Parental and proband SM was assessed using previously identified item sets from the Social Responsiveness Scale (SRS). Children who had parents with low SM scores (less impairments) showed significantly lower impairments in SM compared to children who had either one or both parents with elevated SM scores. No parent-of-origin effect was identified. No significant interactions were found involving proband sex or intellectual disability (ID) status (presence/absence of ID) with paternal or maternal SM. This study establishes that low SM in children with ASD may be driven, in part, by lower SM in one or both parents. Future investigations should utilize larger family pedigrees, including simplex and multiplex families, evaluate other measures of SM, and include other related, yet distinct constructs, such as social inhibition and anhedonia. This will help to gain finer-grained insights into the factors and mechanisms accounting for individual differences in sociability among typically developing children as well as those with, or at risk, for developing ASD.

## Introduction

Social motivation (SM), or the drive to engage, affiliate, and interact with others, has been proposed as a crucial factor for human adaptation and survival throughout evolution (Boyd et al., 2011; Tomasello et al., 2012). Lack or low levels of SM during very early development has been suggested as a key mechanism behind the subsequent social interaction and communication impairments that characterize autism spectrum disorder (ASD) (Chevallier et al., 2012; Kohls et al., 2012). More specifically, it has been hypothesized that due to low SM, children with ASD are less likely to orient to socially salient stimuli that provide key information for learning and the development and specialization of brain circuits underpinning processes crucial for the ability to successfully navigate the complexities of the social world (Mundy, 1995; Dawson et al., 2005). Although the described causal pathway is yet to be confirmed through longer-term longitudinal studies, several lines of evidence provide some support for the SM theory. Firstly, lack of orienting to, and preference for, visual and auditory social stimuli, have been found during early development (Dawson et al., 1998; Osterling et al., 2002; Klin et al., 2009; Falck-Ytter et al., 2013) and throughout later childhood and adolescence (Klin et al., 2002; Sasson et al., 2011; Chevallier et al., 2015; Wright et al., 2016). Secondly, both structural and functional neuroimaging studies have provided consistent evidence for atypicality in key brain regions within the reward processing circuitry (Scott-Van Zeeland et al., 2010; Delmonte et al., 2012; Herrington et al., 2017; Kohls et al., 2018), although it is still unclear whether noted deficits are constrained to social rewards or extend across other reward types (Clements et al., 2018). Importantly, Naturalistic Developmental Behavioral Interventions such as the Early Start Denver Model (ESDM) (Rogers and Dawson, 2010) and Pivotal Response Treatment (PRT) (Koegel et al., 1999) that focus, among other aspects, on SM as a treatment target, have been shown to be effective in improving a range of skills and domains and to result in the need for fewer services later in life (Cidev et al., 2017; Sandbank et al., 2020).

There is pronounced variability in SM among individuals with ASD, with some individuals lacking social interest and awareness of others or actively avoiding social interactions, and others showing the strong drive to form and sustain friendships and romantic relationships and often experiencing loneliness (Wing and Gould, 1979; Bauminger et al., 2008; Calder et al., 2012; Mendelson et al., 2016; Uljarević et al., 2020a). However, despite the centrality of SM in ASD, the factors accounting for large individual differences in this domain remain poorly characterized and understood. Across a range of neurodevelopmental disorders, even in cases of deleterious *de novo* mutations, parental traits have been shown to provide a substantial contribution to the phenotypic variability in children’s morphological, behavioral and cognitive characteristics (Hanson et al., 2014; Moreno De Luca et al., 2015; Klaassen et al., 2016; Evans and Uljarević, 2018). Therefore, consideration of SM among parents of children with ASD might provide a potentially promising means for understanding the sources of individual variability in SM among their children.

The presence of the broader autism phenotype (BAP) among parents and family members of individuals with ASD has been recognized since original clinical descriptions by Kanner (1943). Subsequent studies have provided robust empirical evidence that parents of children with ASD tend to show higher levels of difficulties in language, communication, social interaction, and cognition as well as the presence of certain higher-order repetitive behaviors when compared to the general population (Gerdts and Bernier, 2011; Sucksmith et al., 2011). Importantly, evidence of familiality and inter-generational transmission of these traits has also been reported (Virkud et al., 2009; De la Marche et al., 2012; Taylor et al., 2013; Lyall et al., 2014; Uljarević et al., 2016). Both clinical observations by Kanner (1943) and several studies that focused on personality characteristics (e.g., Bolton et al., 1994; Piven et al., 1997; Bailey et al., 1998) have reported traits indicative of lower levels of SM among parents of children with ASD; however, the pattern of relationship between SM in children with ASD and their parents remains largely unexplored. The only exceptions are a study by Sung et al. (2005) that demonstrated high heritability of SM in a sample of 201 families with a child with ASD and a study by Jones et al. (2017) that reported an association between lower levels of parental SM with shorter peak look at faces in their infant children. However, Sung et al. (2005) used the SM subscale of the Broader Autism Phenotype Scale (Dawson et al., 2007) which consists of only two items, therefore providing limited range. Similarly, Jones et al. (2017) used the Social Competence Questionnaire (Sarason et al., 1985) and the Social Avoidance and Distress Scale (Watson and Friend, 1969) that assess social comfort and social anxiety, respectively, rather than directly assessing SM. In addition to measurement limitations, both studies were limited by small sample size.

The current study aimed to characterize the relationship between parental and proband SM. It was hypothesized that higher levels of SM impairments in parents would be associated with higher levels of SM impairment in their children with ASD. Given the well established sex differences in SM across normative development and neurodevelopmental disorders, including ASD (Sedgewick et al., 2016; Uljarević et al., 2020b, c), we aimed to explore the possibility of sex-specific transmission of SM. Recent findings suggest that familial risk and heritability may vary depending on the presence or absence of intellectual disability (ID) in probands (Xie et al., 2019), therefore, the familiality pattern of SM depending on the IQ status of the child with ASD was investigated. In this study, parent and proband SM was measured by the SM factor derived in our recent analysis of the Social Responsiveness Scale (SRS-2; Constantino and Gruber, 2005, 2012). The SM factor utilized here was derived in a large sample of *N* = 27,953 individuals spanning normative and atypical development, including ASD (Uljarević et al., 2020b). We have opted for this specific SRS-2 subscale over the original SM subscale proposed by Constantino and Gruber (2005, 2012) given that the latter was not supported by any of the SRS/SRS-2 factor analytic investigations (e.g., Frazier et al., 2014; Uljarević et al., 2020b). Factor analysis by Frazier et al. (2014) derived a social avoidance factor that included several items related to SM, however, this factor also contained several items that do not readily map onto the construct of SM (e.g., “Expressions on his/her face don’t match what he/she is saying”, and “Is too tense in social situations”). Therefore, to ensure that several distinct constructs are not conflated within a single factor, we have chosen to focus on the SM scale derived in our work given that it was specifically optimized to capture only that specific construct and excluded any other broad/not-related items.

## Methods

### Participants

Data was obtained from the Simons Simplex Collection (SSC) project. The SSC consisted of a sample of clinically referred individuals with a diagnosis of ASD but without any other medical conditions and their families. Participants were recruited from 12 university-based sites (Fischbach and Lord, 2010). No age restrictions were applied. Data from 2,759 children with ASD (*M*_*age*_ = 9.03 years, SD_*age*_ = 3.57, range: 4–18 years; 375 females) and their parents [*N* = 2,747 fathers (*M*_*age*_ = 42.5 years, SD_*age*_ = 6.4, range: 22–55 years); *N* = 2,752 mothers (*M*_*age*_ = 40.4 years, SD_*age*_ = 5.7, range: 21–58 years)] was included in this study.

### Procedures and Measures

This study was approved by the Stanford University Institutional Review Board. All participants or their parent/legal guardian have provided informed consent for participation as part of SSC.

*The Social Responsiveness Scale* (*SRS*; Constantino and Gruber, 2005, 2012). The SRS is a 65-item measure designed to index autism trait severity. Each item is rated on a 4-point Likert scale (from 1 = Not True to 4 = Almost Always True) with higher scores indicating higher trait severity/atypicality. Mothers and fathers rated their own traits and behaviors using the adult SRS form, and mothers completed a parent-report version of the SRS for their child with ASD. As noted, in this study we utilized the subscale derived in our previous work (Uljarević et al., 2020b) that contains five items and captures SM. Although originally labeled as Attachment and Affiliation to be aligned with the Research Domain Criteria nomenclature that does not specifically highlight SM as a distinct construct, all five items within this factor map onto the SM construct and do not include attachment-related aspects. In this sample, the SM subscale derived in our recent study (Uljarević et al., 2020b) showed good internal consistency in fathers (α = 0.81) and acceptable internal consistency in mothers (α = 0.74) and children with ASD (α = 0.74). We have chosen a five-item SM factor derived in our previous work over the originally proposed, theoretically derived SRS Social Motivation Scale (Constantino and Gruber, 2005, 2012) which has not been replicated in the subsequent factorizations of the SRS and over the Social Avoidance SRS factor derived by Frazier et al. (2014) given that this factor included several items that do not readily map onto SM (e.g., “Expressions on his/her face don’t match what he/she is saying”, and “Is too tense in social situations”).

## Results

**FIGURE 1:**
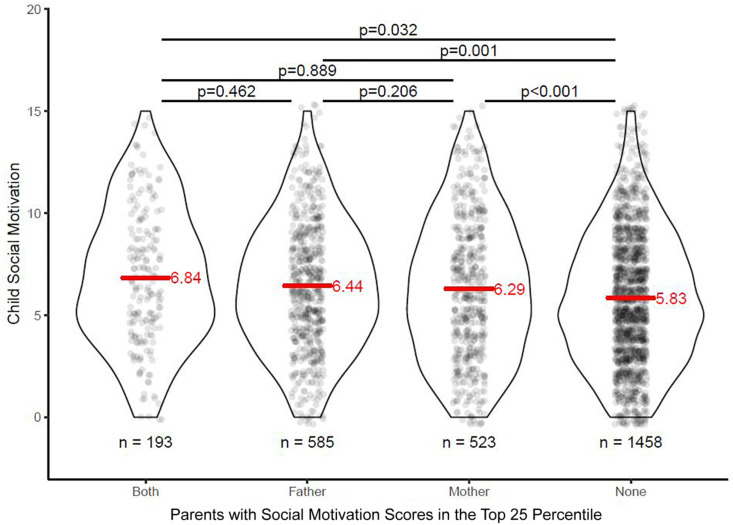
Children’s social motivation scores as a function of parental social motivation status. Both, both mother and father had SM scores in the top 25 percentile of the respective score distribution; neither, neither mother or father had SM scores in the top 25 percentile of the respective score distribution; SM, social motivation.

Effects of parental SM on children’s SM was firstly investigated by conducting a comparison between children whose mother or father had elevated SM scores. Elevated parental SM score was defined as the top 25th percentile of the score distribution for mothers and fathers, respectively, and the remaining distribution was used as the referent group. Children whose parents both reported low personal SM scores (lower impairment) showed significantly lower impairment in SM compared to children who had either one or both parents with elevated SM scores (Figure 1). A cross-tabulation of these dichotomous SM impairment factors for mothers and fathers resulted in four groups (neither parent with elevated SM scores, only mother with elevated SM scores, only father with elevated SM scores, both parents with elevated SM scores). An analysis of variance (ANOVA) on child SM scores showed a significant difference between these groups, *F*(3,2743) = 9.01, *p* < 0.001, and a subsequent Tukey’s *post hoc* test showed that child had significantly poorer SM when either one or both parents had elevated SM scores. However, child SM was not significantly exasperated when both parents had elevated SM scores compared to just one parent. Please see Figure 1 for the score distribution and Table 1 for a detailed overview of the *post hoc* comparisons.

**TABLE 1:** Summary of *post hoc* comparisons.

Contrast	Estimate	95% CI	Adjusted *p*-value
Only father-both	−0.40	−1.11 to 0.3	0.462
Only mother-both	−0.54	−1.26 to 0.17	0.206
Only mother-only father	−0.14	−0.66 to 0.37	0.889
Neither-both	−1.01	−1.66 to −0.36	0.000
Neither-only father	−0.61	−1.02 to −0.19	0.001
Neither-only mother	−0.46	−0.9 to −0.03	0.032

*Both parents, both mother and father had SM scores in the top 25 percentile of the respective score distribution; neither, neither mother or father had SM scores in the top 25 percentile of the respective score distribution; SM, social motivation.*

**FIGURE 2:**
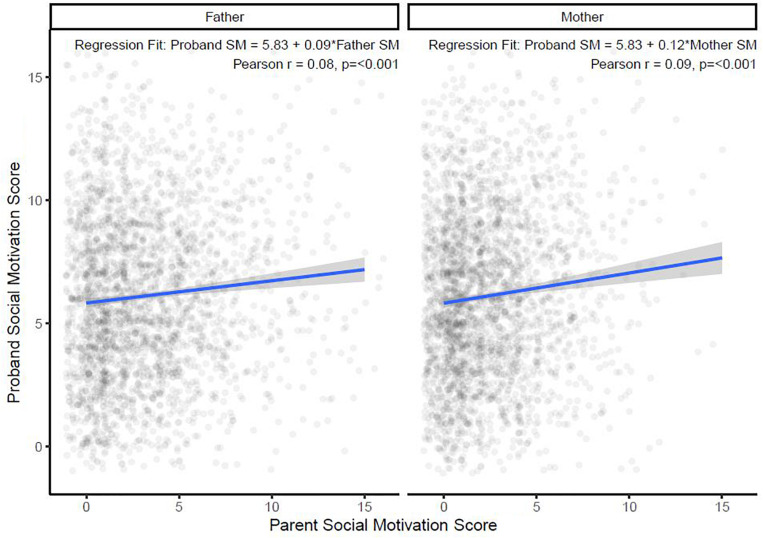
Relationship between paternal and maternal social motivation scores with autistic children’s with social motivation scores. SM, social motivation.

Next, a linear regression model was used to investigate the relationship between SM scores of parents and their child with ASD (Figure 2). An increase of 1 unit in mother SM score was significantly associated with a small increase (0.12; 95% CI: 0.07, 0.17; *p* < 0.001; Model 1, Table 2) in child SM, and the same 1 unit increase in father SM was significantly associated with a similarly small increase (0.09; 95% CI: 0.05, 0.13; *p* < 0.001; Model 2 in Table 2) in child SM. A multivariate regression model was then fitted with child SM as the outcome and both mother and father SM included in the model with an interaction term (Model 3, Table 2). The interaction term was non-significant and therefore dropped from the final model, which showed a cumulative effect of maternal SM (0.12; 95% CI: 0.07, 0.18; *p* < 0.001) and paternal SM (0.09; 95% CI: 0.05, 0.13; *p* < 0.001; Model 4, Table 2) on child SM. Full regression models are presented in Table 2.

**TABLE 2:** Summary of regression models.

	Estimate	95% CI	*F*	*t*	*p*	*R*^2^
**Model 1**			22.87			0.008
SM mother	0.12	0.07 to 0.017		4.78	<0.001	
**Model 2**			19.65			0.007
SM father	0.09	0.05 to 0.13		4.43	< 0.001	
**Model 3**			14.55			0.015
SM mother	0.13	0.06 to 0.20		3.62	<0.001	
SM father	0.10	0.04 to 0.15		3.53	<0.001	
SM mother × SM father	−0.00	−0.02 to 0.01		−0.31	0.757	
**Model 4**			21.79			0.015
SM mother	0.12	0.07 to 0.18		4.85	<0.001	
SM father	0.09	0.05 to 0.13		4.58	<0.001	
**Model 5**			7.77			0.007
SM mother	0.13	−0.00 to 0.26		1.96	0.051	
Proband sex	−0.09	−0.61 to 0.42		−0.35	0.728	
SM Mother × proband sex	−0.01	−0.15 to 0.13		−0.16	0.876	
**Model 6**			7.21			0.007
SM father	0.03	−0.08 to 0.14		0.55	0.582	
Proband sex	−0.37	−0.89 to 0.15		−1.39	0.165	
SM father × proband sex	0.07	−0.05 to 0.19		1.17	0.244	
**Model 7**			29.19			0.030
SM mother	0.10	0.01 to 0.20		2.11	0.035	
Proband ID	−1.17	−1.54 to −0.80		−6.16	<0.001	
SM mother × proband ID	0.03	−0.08 to 0.15		0.59	0.553	
**Model 8**			29.96			0.031
SM father	0.05	−0.03 to 0.13		1.18	0.238	
Proband ID	−1.35	−1.73 to −0.97		−6.98	<0.001	
SM father × proband ID	0.08	−0.01 to 0.17		1.71	0.088	
**Model 9**			28.72			0.039
SM mother	0.13	0.08 to 0.18		5.14	<0.001	
SM father	0.11	0.07 to 0.15		5.45	<0.001	
Proband sex	0.02	−0.34 to 0.38		0.13	0.9	
Proband ID	−1.14	−1.41 to −0.88		−8.33	<0.001	

*CI, confidence intervals; ID, presence/absence of intellectual disability; SM, social motivation.*

**FIGURE 3:**
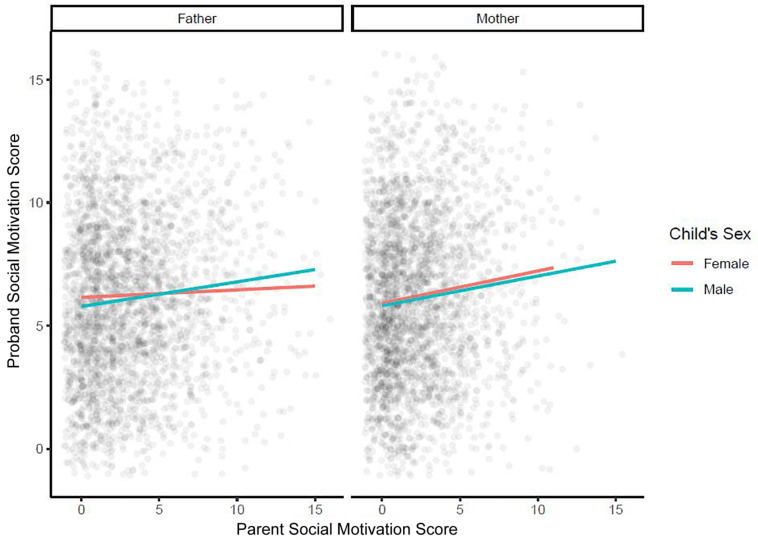
Multiple regression model examining whether the effect of each parent’s social motivation on a child’s social motivation depends on a child’s sex. SM, social motivation.

**FIGURE 4:**
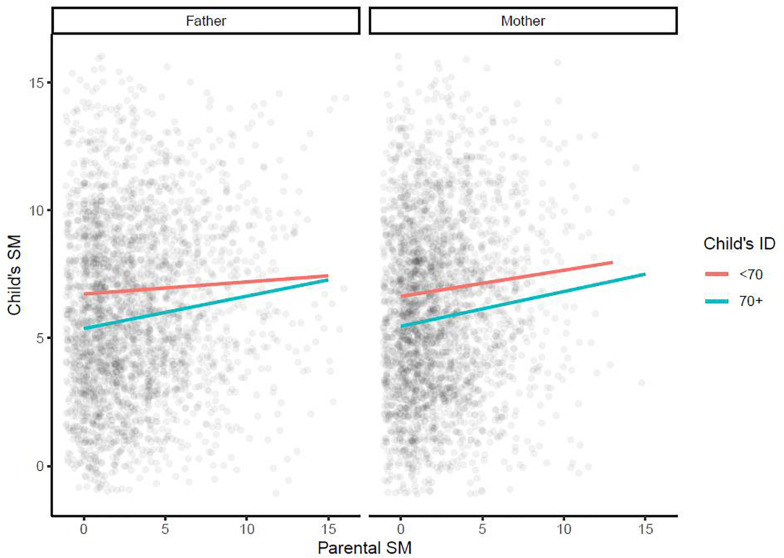
Multiple regression model examining whether the effect of each parent’s social motivation on a child’s social motivation depends on the presence/absence of intellectual disability in children. ID, intellectual disability; SM, social motivation.

Further multiple regression models were used to examine whether the effect of each parent’s SM on the child’s SM depended on the child’s sex [Figure 3; models 5 (effect of maternal SM) and 6 (effect of paternal SM) in Table 2] and/or on the presence/absence of ID in the child [Figure 4; models 7 (effect of maternal SM) and 8 (effect of paternal SM) in Table 2]. No significant sex interaction with paternal or maternal SM was found. The observed paternal effect on a male child was over threefold higher than on a female child, however, it was not statistically significant. No significant IQ interaction with paternal or maternal SM was found. A final multiple regression model was run with mother SM, father SM, child sex, and child ID as covariates (Model 9, Table 2). Mother SM (0.13; 95% CI: 0.08, 0.18; *p* < 0.001), father SM (0.11; 95% CI: 0.07, 0.15; *p* < 0.001) and ID (−1.14; 95% CI: −1.41, −0.88, *p* < 0.001) were all significant predictors of child SM, while sex (male: 0.02, 95% CI: −0.34, 0.38, *p* = 0.9) was not significant as a predictor. Full regression models are presented in Table 2.

## Discussion

The current study aimed to examine the familiality of SM by exploring the link between parental and proband the social responsiveness scale (SRS-2) SM scores. Our analysis demonstrated that low levels of paternal and maternal SM were associated with a significant deficit in SM in children with ASD. Importantly, these effects were independent and cumulative, and no parent-of-origin effect was found. This finding is in line with two previous studies that have investigated familiality and heritability of SM in small samples of families of children with ASD (Sung et al., 2005) and those with typically developing youth (Jones et al., 2017). While indications for potential sex-specific transmission of SM were observed as paternal effect on a male child with ASD was over three-fold higher than the effect on a female child with ASD, this effect was no statistically significant and these findings should therefore be interpreted as very preliminary and warrant further replication.

The present study used the SSC data which is a relatively large and well-characterized sample of mother-father-child with ASD triads. In contrast to previous studies by Sung et al. (2005) and Jones et al. (2017) who used a two-item subscale and constructs of social discomfort and anxiety to capture SM, respectively, our investigation utilized SM items derived from the SRS in our recent SRS factorization (Uljarević et al., 2020b). The SM scale used here had good conceptual clarity as it encompasses only items directly relating to the drive for social approach/to interact socially. However, the findings reported here should also be considered in light of several limitations. Firstly, we relied on a questionnaire measure of SM and therefore a potential impact of the common method variance will need to be considered. This is particularly relevant in the light of the findings by De la Marche et al. (2015) and Jones et al. (2017) that emphasize potential method-specific (questionnaire versus more objective assessments and experimental protocols) pattern of findings in the studies of similar design as ours. Therefore, it will be crucial to replicate and further refine findings reported here by utilizing multi-method assessment protocols. Secondly, the sample used here only included simplex families and did not include a general population sample. Given the suggestions that etiologic mechanisms operating within simplex and multiplex families might be somewhat distinct (Virkud et al., 2009; Lyall et al., 2014), it will be important for future studies to better characterize the pattern of transmission of SM depending on simplex versus multiplex status and whether any potential specificities would emerge when compared to the transmission pattern in the general population. Thirdly, although SSC database afforded a significantly larger sample size for female participants. However, given the well established over-representation of ASD in males, the sample used in this study was nevertheless heavily skewed toward male participants, which could have impacted the ability to detect some of the more nuanced sex-specific effects. Therefore, it will be important for future studies to further investigate the possibility of sex-specific transmission of SM.

Importantly, SM is a complex construct and has been suggested to encompass a range of inter-related elements including social orienting, seeking enjoyment in social interactions, and behaviors and actions aimed at maintaining social bonds (Chevallier et al., 2012). The SM scale used here only captures the seeking/enjoyment element, and it is not clear whether the familiality pattern would be continuous with the social orienting and maintenance elements, or whether potential discontinuities might arise. Despite the centrality of the SM construct in ASD, there is a paucity of instruments that can effectively and comprehensively capture individual differences in SM in a sensitive and quantitative manner. The recently developed Stanford Social Dimensions Scale (SSDS) (Phillips et al., 2019) has been specifically designed to capture a broad spectrum of traits and behaviors indicative of the seeking/linking and maintenance components described by Chevallier et al. (2012) and shows promising psychometric properties and ability to capture individual differences in distinct SM subdomains in children and adolescents with ASD (Uljarević et al., 2020a). It will therefore be crucial for future investigations to incorporate the SSDS and other scales capturing related, yet distinct constructs such as social inhibition and anhedonia, to gain an in-depth insight into the factors and mechanisms accounting for the individual differences in key determinants of sociability among children with, and at risk, for developing ASD.

## Data Availability Statement

Publicly available datasets were analyzed in this study. This data can be found here: www.sfari.org.

## Ethics Statement

The studies involving human participants were reviewed and approved by the Stanford University Institutional Review Board. Written informed consent to participate in this study was provided by the participants’ legal guardian/next of kin.

## Author Contributions

MU, TF, and AH designed the study. MU, WB, and MC analyzed the data. MU, TF, BJ, JP, WB, and AH wrote the manuscript. All authors reviewed the manuscript and approved the final version.

## Conflict of Interest

The authors declare that the research was conducted in the absence of any commercial or financial relationships that could be construed as a potential conflict of interest.
